# Production of anti-inflammatory and antidiabetic oligosaccharides from okra mucilage through one-step microbial fermentation

**DOI:** 10.1371/journal.pone.0351134

**Published:** 2026-06-11

**Authors:** Ayaz Ahmad, Imdad Ullah Khan, Yusra Jamil, Aymen Ajmal, Murad Ali, Muhammad Ihtesham, Douglas Law, Patricio R. De los Ríos-Escalante, Saeedah Almutairi, Wahidah H. Al-Qahtani, Ahmed Othman Alsabih, Mostafa A. Abdel-Maksoud, Waheed Ahmad

**Affiliations:** 1 Department of Biotechnology, Abdul Wali Khan University Mardan, Mardan, Khyber Pakhtunkhwa, Pakistan; 2 Faculty of Health and Life Sciences, INTI International University, Nilai, Negeri Sembilan, Malaysia; 3 Núcleo de Estudios Ambientales, Facultad de Recursos Naturales, Universidad Católica de Temuco, Temuco, Chile; 4 Departamento de Ciencias Biológicas y Químicas, Facultad de Recursos Naturales, Universidad Católica de Temuco, Temuco, Chile; 5 Botany and Microbiology Department, College of Science, King Saud University, Riyadh, Saudi Arabia; 6 Department of Food Sciences & Nutrition, College of Food and Agricultural Sciences, King Saud University, Riyadh, Saudi Arabia; 7 Department of Physiology, College of Medicine, King Saud University, Riyadh, Saudi Arabia; 8 Research Chair of Biomedical Applications of Nanomaterials, Biochemistry Department, College of Science, King Saud University, Riyadh, Saudi Arabia; 9 Minnesota Dental Research Center for Biomaterials and Biomechanics, School of Dentistry, University of Minnesota, Minneapolis, Minnesota, United States of America; Kafr el-Sheikh University: Kafrelsheikh University, EGYPT

## Abstract

Plant-based natural products are increasingly being explored as safer and more sustainable alternatives than synthetic drugs for controlling globally rising chronic health conditions, including inflammation and diabetes. Microbial fermentation has long been used to enhance the value and bioactivity of natural products. In this study, we produced bioactive oligosaccharides from okra (*Abelmoschus esculentus*) mucilage polysaccharides using a sustainable one-step *Pichia kudriavzevii*-mediated fermentation approach. The oligosaccharide fractions separated by size-exclusion chromatography primarily contained uronic acids, pentoses, and hexoses. In vitro assays showed that these oligosaccharides possess notable anti-inflammatory and antidiabetic potential. Among the fractions, ODF91 exhibited the strongest membrane-stabilizing activity, providing 92.86% protection of the erythrocyte membrane, comparable to that of the standard anti-inflammatory drug. ODF91, at 200 mg/kg, markedly reduced paw edema by 94.69% in the carrageenan-induced mouse model, supporting preliminary in vivo anti-inflammatory activity. ODF91, at 200 mg/kg, also lowered blood glucose levels in streptozotocin-induced diabetic mice by 86.91%, followed by ODF83 and ODF84, suggesting promising preliminary antihyperglycemic activity. Furthermore, lipid profile parameters significantly improved in diabetic mice after treatment with these oligosaccharides. Collectively, these results suggest that the fermented okra-derived oligosaccharides possess promising anti-inflammatory and antidiabetic properties and highlight microbial fermentation as a scalable strategy for converting underutilized plant polysaccharides into value-added bioactive products.

## 1. Introduction

The ever-growing global burden of chronic diseases, particularly inflammatory disorders and diabetes mellitus, is a motivation for finding successful, safe, and sustainable therapeutic strategies. Chronic inflammation is responsible for the development and progression of various serious clinical disorders, such as cardiovascular diseases (CVDs), arthritis, and cancer [1]. Although synthetic anti-inflammatory medicines remain a mainstay of treatment, their prolonged use is linked to serious adverse drug reactions [2]. On the same note, the treatment of type 2 diabetes, a pervasive metabolic syndrome mediated by insulin resistance and hyperglycemia, relies mainly on pharmacological agents that fail to halt disease progression, and their long-term use incurs adverse side effects [3]. This landscape has led to a paradigm shift towards searching for bioactive natural products as sustainable alternatives that help manage disease progression and have no or fewer off-target effects [4].

In this search, plant-derived polysaccharides have emerged as promising sources of biocompatible and multifunctional compounds. In particular, polysaccharides obtained from plant mucilage plants have been of interest due to their reported biological activities, including antioxidant and anti-inflammatory potential [5,6]. The Abelmoschus esculentus (okra) mucilage is a complex heteropolysaccharide rich in D-galactose, L-rhamnose, and galacturonic acid, which has been reported to have antitumor, antimicrobial, and hypoglycemic effects [7,8]. Nevertheless, the native high molecular weight of these polymers may limit their bioavailability and bioactivity, a major bottleneck in their therapeutic usage.

To address these limitations, microbial fermentation has garnered immense attention as an effective, green bioprocessing technique. This strategy selectively depolymerizes polysaccharides into more bioactive oligosaccharides and, in many cases, improves or unveils new bioactivities without the use of harsh chemicals [9]. For instance, the fermentation of seaweed polysaccharides by Lactobacillus spp. has been reported to increase the glucuronic acid content and decrease the molecular weight, thereby enhancing bioactivity. The technique leverages the specificity of microbial enzymes, reduces the formation of by-products, and aligns with the principles of sustainable production [10]. Despite these advantages, the microbial transformation of okra mucilage polysaccharides and the therapeutic potential of the produced oligosaccharides remain underexplored. We hypothesized that *P. kudriavzevii*-mediated fermentation would depolymerize okra mucilage polysaccharides into lower-molecular-weight oligosaccharide fractions with improved biological activity. *P. kudriavzevii* was selected because of its robustness under acidic fermentation conditions, tolerance to variable substrate environments, and ability to utilize carbohydrate-rich plant materials, making it suitable for green bioprocessing of okra mucilage polysaccharides.

Although previous studies have reported biological activities of crude okra polysaccharides and fermentation-modified plant polysaccharides, limited information is available on the one-step microbial conversion of okra mucilage polysaccharides into fractionated bioactive oligosaccharides. The present study is distinct because it combines *P. kudriavzevii*-mediated fermentation, size-exclusion fractionation, compositional profiling, and integrated in vitro and in vivo evaluation of anti-inflammatory and antidiabetic activities. Therefore, this work provides preliminary evidence for a green bioprocessing strategy to generate functional oligosaccharide fractions from okra mucilage.

## 2. Materials and methods

### 2.1. Approval of the study

This study was recommended by the Graduate Study Committee (GSC) of the Department of Biotechnology, Abdul Wali Khan University Mardan (AWKUM), Pakistan, in the 13^th^ meeting, approved by the Sub-ASRB (Sub-Advanced Study and Research Board of the Faculty of Chemical and Life Sciences, AWKUM, Pakistan) in the 7^th^ meeting held on April 26, 2023 and notified vide # Dir A&R/AWKUM/2023/9387.

### 2.2. Inclusivity in global research

This study was conducted in Pakistan with the direct involvement of local researchers from Abdul Wali Khan University Mardan, in the study design, experimental work, data analysis, interpretation, and manuscript preparation. The okra material used in this study was purchased from a local commercial market in Mardan, Pakistan, and no indigenous, protected, archaeological, culturally significant, or wild-collected materials were used. Human red blood cells were obtained from a donor after written informed consent, and all animal experiments were performed following institutional ethical approval. Additional information regarding the ethical, cultural, and scientific considerations specific to inclusivity in global research is provided in the Supporting Information (S1 Checklist).

### 2.3. Polysaccharides extraction

Fresh okra pods were obtained from a commercial market in the district of Mardan, Pakistan, during the harvesting season and were manually screened. The unwanted parts and materials were removed. The okra pods and seeds were disinfected by soaking for 20 minutes in a 5% sodium hypochlorite solution (containing 4–6% active chlorine) and rinsed several times with deionized distilled water to remove residual sodium hypochlorite (DAEJUNG) traces. The okra pods and seeds were then immersed in autoclaved distilled water to extract mucilage, following the protocol prescribed earlier [11], with some modifications. 100 g of disinfected okra pods and seeds were immersed in 1 L of distilled water at 24 ± 2 °C for 24 h. The aqueous extract was then filtered through muslin cloth, and polysaccharides were precipitated by overnight treatment with 75% ethanol (Sigma Aldrich). The resulting ethanol-precipitated polysaccharides (EPP) were pelleted by centrifugation at 4500 × g for 10 min at 4 °C and freeze-dried subsequently.

### 2.4. One-step microbial digestion

Free inoculum of *Pichia kudriavzevii* was maintained in YPD (yeast extract, peptone, and dextrose) (Sigma Aldrich) medium for 24–48 h. *P. kudriavzevii* cells were enumerated physically by means of a Neubauer chamber using a light microscope [12]. Before inoculation, yeast cells were harvested and washed twice with sterile distilled water to remove residual YPD medium components. The fermentation medium was inoculated with 1 × 10^5^ yeast cells/flask and supplemented with 1% (w/v) EPP derived from okra pods and seed mucilage, along with 1 g/L ammonium chloride as the sole nitrogen source. EPP served as the sole added carbon source in the fermentation medium. The fermentation was carried out in 100 mL medium, adjusted to an initial pH of 5.8, and maintained under continuous shaking at 28 °C with agitation at 160 rpm for 48 h. Yeast proliferation was monitored regularly (0-48h) by measuring turbidity at 600 nm with a UV-visible spectrophotometer and by viable cell counts, expressed as log_10_CFU/mL, using the standard plate count method. The pH of the medium was not controlled; it naturally changed over time. Upon completion of 48 h of fermentation, yeast biomass was purified by centrifugation at 4,500 × g for 30 min, and the supernatant was lyophilized and processed for oligosaccharide purification.

### 2.5. Size exclusion chromatography

The fermented product was fractionated by size-exclusion chromatography (SEC). The size-exclusion chromatography column was packed with Bio Gel P10 (Bio-Rad, Catalogue: 1504144), which has an approximate fractionation range of 1,500–20,000 Da; the reported 45–90 µm range refers to bead size rather than pore size. The column was equilibrated before sample loading [13]. Lyophilized oligosaccharides were re-dissolved at 0.5% w/v and loaded into the column at 5% v/v ratio. The SEC column (2.8 cm × 40 cm) was already calibrated with dextran (Sigma Aldrich). Autoclaved distilled water served as the eluent, and 2 mL of each fraction was collected into Eppendorf tubes at a flow rate of 0.3 mL/min. Collected fractions were monitored by the phenol–sulfuric acid assay, and absorbance was measured at 490 nm to identify carbohydrate-containing fractions.

### 2.6. Biochemical analysis

The biochemical analysis of the obtained oligosaccharide fractions was assessed as reported earlier. The total carbohydrate content was quantified using the phenol-sulfuric acid (PSA) method [14]. Uronic acid contents were measured by the m-hydroxyl biphenyl method [15], while the unsaturated uronic acid contents were calculated by using the thiobarbituric (TBA) method [16]. Similarly, the hexose content in each fraction was determined using the anthrone reagent and the pentose content using orcinol solution with 1% iron chloride solution [17].

### 2.7. Hemolytic assay

The cytotoxicity of digested oligosaccharide fractions was estimated using human red blood cells (RBCs), obtained from a donor after informed written consent on December 13, 2023 [18]. The collection and use of human blood and all animal handling procedures were approved by the Institutional Animal Care and Ethics Committee of the Department of Biotechnology, Abdul Wali Khan University, Mardan, Pakistan, in compliance with the Declaration of Helsinki (Ethical Approval Certificate: AWKUM/Biotech/2023/159). RBCs were isolated from 5 mL of fresh blood through centrifugation at 1500 rpm for 5 min. The RBCs were rinsed and resuspended in PBS at a 1:3 ratio. The RBC suspension was incubated with selected fractions at varying concentrations (50 µg/mL, 100 µg/mL, 200 µg/mL, and 400 µg/mL) and maintained at 37 °C for 1 h. Upon completion of the incubation period, the reaction was centrifuged, and the optical density of the supernatant was recorded at 570 nm employing an ELISA BioTek plate reader (Agilent Technologies, Model: Elx 800). Triton-X 100 at 0.5% served as a positive control. The following equation estimated the percent hemolysis of each fraction:


$$\begin{array}{c} Percent\;Hemolysis=\frac{mean\;OD\;of\;Sample-mean\;OD\;of\;PBS}{mean\;OD\;of\;positive\;control-mean\;OD\;of\;PBS}*\text{1}\text{0}\text{0} \end{array}$$

### 2.8. Erythrocyte membrane stabilization assay

The in vitro anti-inflammatory potential of oligosaccharides obtained from *P. kudriavzevii* fermented okra pod and seed mucilage was assessed using the erythrocyte membrane stabilization assay [19]. Human red blood cells (RBCs) were freshly collected from healthy donors (after written consent) on December 13, 2023, and prepared as a 40% suspension. 0.1 mL of RBCs was mixed with a hypotonic solution containing oligosaccharides at different concentrations (50 µg/mL, 100 µg/mL, 200 µg/mL, and 400 µg/mL). The reaction mix was maintained at 25 °C for 1 h and centrifuged at 2000 rpm for 10 min. The absorbance of the supernatant was measured at 540 nm. Indomethacin served as a standard anti-inflammatory drug. The percent membrane stabilization was estimated through the following standard formula:


$$\begin{array}{c} Percent\;Inhibtion\;(\%)=\frac{mean\;Abs\;of\;A\text{1}-mean\;Abs\;of\;A\text{2}}{mean\;Abs\;of\;A\text{3}-mean\;Abs\;of\;A\text{2}}*\text{1}\text{0}\text{0} \end{array}$$

Where A1 refers to the mean absorbance of the fraction in a hypotonic solution.

A2 refers to the mean absorbance of the fraction in an isotonic solution.

A3 refers to the mean absorbance of the positive control in a hypotonic solution.

### 2.9. Carrageenan-induced paw edema

The in vivo anti-inflammatory activity of the polysaccharide fractions was evaluated using a well-established protocol in Balb/C mice [20]. Ethical approval for all animal studies was obtained from the Institutional Animal Care and Ethics Committee of the Department of Biotechnology, Abdul Wali Khan University Mardan, Pakistan (Certificate No. AWKUM/Biotech/2023/159). The Balb/C mice were sourced from the Veterinary Research Institute (VRI) in Peshawar and underwent a one-week acclimatization period in a controlled environment with a 12-hour light/dark cycle. Animals had unrestricted access to water and a standard diet throughout the study. Animals were monitored daily for general health, behavior, grooming, mobility, food and water intake, and signs of pain or distress. During carrageenan injection and paw-volume measurement, mice were gently restrained to minimize handling stress. No general anesthesia was used during carrageenan injection or paw-volume measurement because the procedures were brief, and analgesics were not routinely administered because they could interfere with the inflammatory response being evaluated. Humane endpoints included severe or persistent distress, abnormal posture, impaired mobility, labored breathing, self-mutilation, or inability to access food or water. No death was used as an experimental endpoint.

To investigate the anti-inflammatory effects of each polysaccharide fraction, mice were divided into 6 groups, with five mice per group. One group served as the normal control, while inflammation was induced in the remaining groups by injecting 1% carrageenan (Sigma-Aldrich) into the right hind paw. After 1 hour, paw edema was allowed to develop, and paw volume was measured using a calibrated screw gauge at 1, 2, 3, and 4 hours post-treatment. Group 1 received no treatment and served as the negative control, whereas group 2 received 150 mg/kg of diclofenac sodium as a positive control. The remaining groups were administered 50, 100, and 200 mg/kg doses of the polysaccharide fractions. Paw edema reduction was calculated by comparing volumes at each time point to baseline measurements, and the percent reduction was determined for each group. At the end of the experiment, mice were humanely euthanized by gradual-fill CO₂ inhalation at a displacement rate of 30–70% of the chamber volume per minute, followed by cervical dislocation to confirm death. Carcasses were disposed of through the institutional biomedical/animal waste disposal system in accordance with relevant institutional biosafety and animal welfare regulations.

### 2.10. Alpha-amylase inhibition assay

The antidiabetic potential of the oligosaccharide fractions was assessed in vitro using the α-amylase inhibitory assay, as described earlier [21]. In this assay, 250 µL of α-amylase enzyme solution (0.5 mg/mL) from (bacterial source, EC: 3.2.2.1, catalogue: A0444-G), prepared in sodium phosphate buffer (Oxoid), was mixed with 50, 100, 200, and 400 µg/mL concentrations of the oligosaccharide fractions. After adding 1% starch solution, the reaction was allowed to proceed at 25 ± 2 °C for 10 minutes. The reaction was then halted by adding 500 µL of dinitrosalicylic acid (DNS), followed by a 5-minute incubation in a hot water bath. The mixture was cooled to room temperature, and the optical density was measured at 540 nm. Acarbose was used as the positive control. The percentage of α-amylase inhibition was calculated using a standard equation as reported previously [21].


$$\begin{array}{c} \%\;Inhibition:\;\frac{As-Ac\;}{Ac}*\text{1}\text{0}\text{0} \end{array}$$

Where:

$A_{c}$= Absorbance of control

$A_{s}$= Absorbance of sample

### 2.11. Alpha-glucosidase inhibition assay

The inhibitory effect of the oligosaccharide fractions on α-glucosidase was measured using a method described previously [21]. Briefly, 100 µL of α-glucosidase enzyme was pre-incubated with 50 µL of the oligosaccharide fractions at concentrations of 50, 100, 200, and 400 µg/mL. The reaction was initiated by adding 50 µL of 3.0 mM p-nitrophenyl-α-D-glucopyranoside, dissolved in 20 mM phosphate buffer, and incubating the mixture at 37 °C for 20 minutes. After the incubation period, the reaction was terminated by adding 0.1 M sodium carbonate (Na_2_CO_3_). The absorbance of the samples was measured at 405 nm, with distilled water used as the blank and acarbose as the reference drug. The percentage of α-glucosidase inhibition was calculated using the formula reported earlier [21].


$$\begin{array}{c} \%\;Inhibition:\;\frac{As-Ac\;}{Ac}*\text{1}\text{0}\text{0} \end{array}$$

Where:

$A_{c}$= Absorbance of control

$A_{s}$= Absorbance of sample

### 2.12. Yeast glucose uptake assay

*Saccharomyces cerevisiae* ATCC 9763, supplied by JK Enterprises, Pakistan, was used to determine the inhibition of okra mucilage EPP and its digested fractions on the glucose (Sigma Aldrich) uptake ability of yeast cells, in accordance with the earlier published protocol [22]. The assay was started by preparing a 1% solution of commercially available bakery yeast in autoclaved distilled water. The yeast cells were purified by centrifugation at 4200 rpm for about 5 min and rinsed repeatedly until a transparent supernatant was obtained. The pellet comprising yeast cells was dissolved in 9 parts of distilled water, yielding a 10% yeast cell suspension. Okra mucilage EPP and its fractions were mixed with 1 mL of a glucose solution at four concentrations (50, 100, 200, and 400 µg/mL) and incubated at 37 °C for 10 min. 100 µL of a 10% yeast inoculum was poured into each concentration. The test mixture was gently homogenized and placed at 37 °C for 60 min. Upon completion of the incubation period, the samples were centrifuged at 2500 rpm for 5 min, and glucose was estimated in the supernatant at 540 nm utilizing a BioTek ELISA plate reader. The percent glucose uptake was evaluated through the published equation [23].


$$\begin{array}{c} \%\;Glucose\;uptake\;:\;\frac{Glucose\;initial-Glucose\;final}{Glucose\;initial}*\text{1}\text{0}\text{0} \end{array}$$

Where:

*Glucose initial* = initial glucose concentration

*Glucose final* = remaining glucose after incubation

### 2.13. Streptozotocin-induced antidiabetic assay

The Institutional Animal Care and Ethics Committee of the Department of Biotechnology, Abdul Wali Khan University Mardan, Pakistan (Approval No. AWKUM/Biotech/2023/159) approved all animal experiments. All the procedures were performed in accordance with the institutional guidelines of laboratory animal care and use. Thirty-six Balb/C mice were obtained from the Veterinary Research Institute (VRI), Peshawar. They were randomly allocated into six groups and acclimatized for a week. After acclimatization, Group I was maintained on a normal diet throughout the experimental period, while diabetes was induced in the remaining groups using intraperitoneal injection of streptozotocin (STZ; 100 mg/kg), freshly dissolved in cold citrate buffer (0.1 M, pH 4.5) immediately before administration.

Animals were monitored daily throughout the study for general health, behavior, grooming, mobility, food and water intake, body weight changes, dehydration, and signs of pain or distress. Humane endpoints included severe or persistent distress, marked lethargy, abnormal posture, labored breathing, inability to eat or drink, severe dehydration, or excessive body-weight loss. No death was used as an experimental endpoint. No general anesthesia was used for STZ injection, oral gavage, or routine glucose monitoring because these procedures were brief; however, animals were gently handled, and analgesics were not routinely administered because they could interfere with the diabetic and metabolic outcomes being evaluated.

After 12 h fasting, fasting blood glucose levels (FBGLs) of normal and diabetic mice were measured at 96 h after STZ injection using an Accu-Chek Performa (Roche Diagnostics, Mannheim, Germany) glucose meter. Mice exhibiting FBGL greater than 11.1 mmol/L were considered diabetic and randomly divided into experimental groups. Each group consisted of six mice (n = 6), and animals were randomly allocated to minimize selection bias. Blinding of outcome assessment and a priori power analysis were not formally implemented in this exploratory study and are acknowledged as limitations. Group II was left untreated, which acted as diabetic control, while Group III received 100 mg/kg of glibenclamide as a standard antidiabetic drug. All experimental mice were administered by oral gavage once a day.

At the end of the treatment, the animals were fasted for 12 h, and body weight and FBGL were recorded. For orbital sinus blood collection, mice were anesthetized with isoflurane inhalation using 3–4% for induction and 1–2% for maintenance in oxygen. Blood samples were drawn from the orbital sinus of each mouse under anesthesia. Serum was separated from whole blood by centrifugation at 5000 rpm for 5 min at 4 °C [24]. The parameters of serum lipid profile, such as total cholesterol (TC), triglycerides (TG), high-density lipoprotein (HDL), and low-density lipoprotein (LDL), were determined using commercially available diagnostic kits in accordance with the manufacturer’s instructions. At the end of the study, mice were humanely euthanized by gradual-fill CO₂ inhalation at a displacement rate of 30–70% of the chamber volume per minute, followed by cervical dislocation to confirm death. Carcasses were disposed of through the institutional biomedical/animal waste disposal system in accordance with relevant institutional biosafety and animal welfare regulations.

### 2.14. Statistical analyses

In vitro experiments were performed in triplicate (n = 3), and the results are presented as mean ± standard deviation (SD). Five animals per group (n = 5) were used in the in vivo experiments. The statistical analysis was done in SPSS version 22. Single-point comparisons were done using one-way ANOVA and post hoc test (Tukey). For time-dependent data, such as paw edema and blood glucose levels, two-way ANOVA (time x treatment) and the Bonferroni post hoc test were used. A p-value below 0.05 was deemed to be statistically significant.

## 3. Results and discussion

### 3.1. *Pichia kudriavzevii*-mediated fermentation of okra polysaccharides

**Fig 1:**
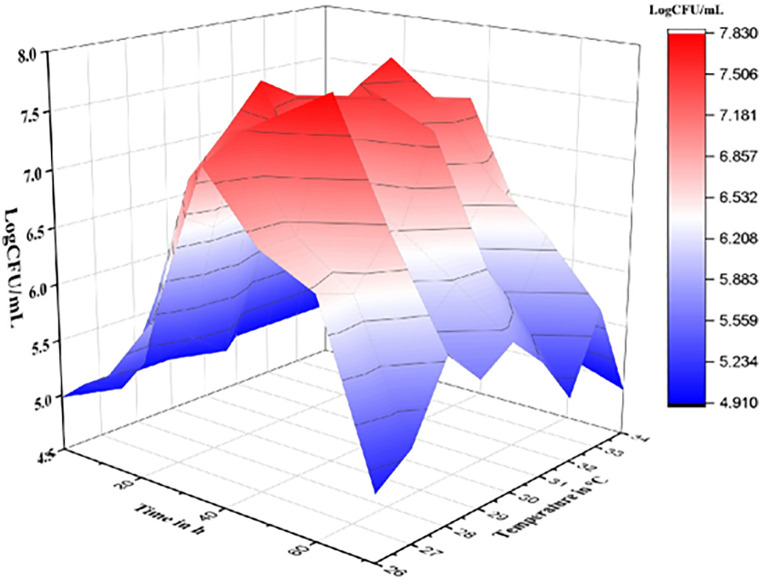
3D surface plot representing the *Pichia kudriavzevii* cell biomass in log_10_CFU/mL under varying temperatures and time conditions (hours) during fermentation.

Polysaccharides are usually hydrolyzed to oligosaccharides using chemical and enzymatic methods. Chemical digestion has several advantages, such as being cheap and simple, but it also has shortcomings. The resulting product is usually very complex, posing a significant challenge for further separation and isolation. In addition, chemical methods may lead to contamination, posing serious environmental hazards [25]. Nevertheless, enzymatic fermentation of polysaccharides offers a promising alternative owing to its higher specificity and moderate reaction conditions. It also has certain disadvantages, such as strict storage conditions, reduced enzyme activity, difficulty in disposal, and high cost, which do not allow its large-scale application for oligosaccharide production [26]. Alternatively, microbial fermentation is a one-step, cost-effective, and sustainable method for the mass production of biologically potent oligosaccharides. *Pichia kudriavzevii* is a non-methylotrophic, stress-tolerant yeast that has been reported to grow on diverse carbohydrate-rich substrates and is increasingly explored for food and bioprocessing applications. It has been of interest as a highly effective host for the production of many industrial products, including recombinant proteins, enzymes, such as trypsin and proteinase K, phospholipase C, and phytase [27]. In this study, 100 g of okra pods and seeds, disinfected with sodium hypochlorite (NaOCl), were immersed in sterile distilled water, which produced 18.23 g of mucilage. The obtained mucilage was freeze-dried and precipitated using 75% ethanol. These polysaccharides were then fermented to oligosaccharides using *P. kudriavzevii*. The fermentation conditions were optimized to achieve the highest possible yield by systematically adjusting one factor at a time. Spectroscopic data were recorded and analyzed to monitor cell density across the exponential, stationary, and decline phases of the fermentation process. Fig 1 shows that *P. kudriavzevii* attained the highest growth, with a cell density of 7.8 log_10_CFU/mL when incubated at 28 °C, pH 5.8, for 48 h with 160 rpm agitation in a shaking incubator. In a previous study, P. pastoris-mediated fermentation of cress polysaccharides showed an increase in cell density from 4.98 ± 0.13 log_10_CFU/mL at 0 h to 7.76 ± 0.19 log_10_CFU/mL at 6 h, and further increase to 8.76 ± 0.17 log_10_CFU/mL at 24 h [28]. Here, a progressive increase in growth between 24 and 48 h; however, when the incubation period exceeds, a decline in growth is observed. Beyond 48 h and at temperatures above 32 °C, growth decreased to 5–5.5 log_10_CFU/mL, likely due to nutrient depletion. These results suggest that incubation at 28 °C and pH 5.8 for 48 h provides the optimal conditions for *P. kudriavzevii*-mediated fermentation and production of bioactive oligosaccharides.

### 3.2. Total carbohydrate contents of the oligosaccharide fractions

**Fig 2:**
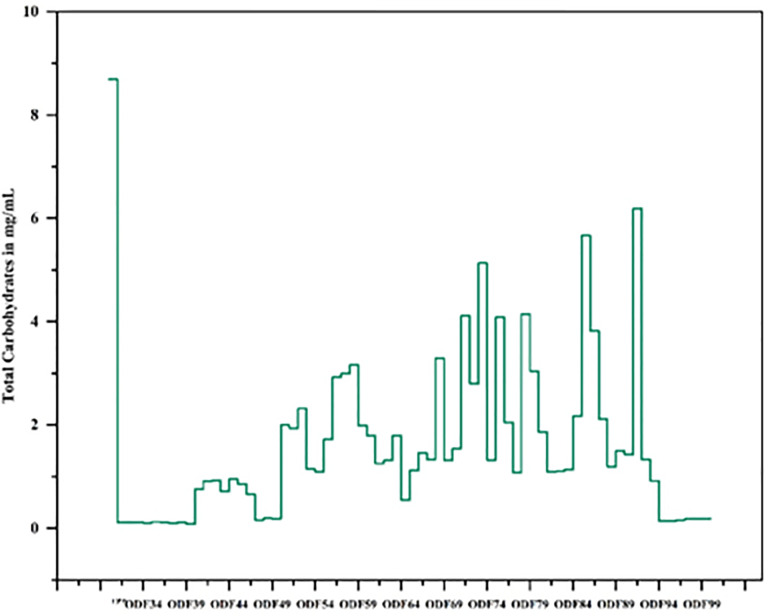
Total carbohydrate contents (mg/mL) of ethanol-precipitated polysaccharide (EPP) and oligosaccharide fractions (ODF34-ODF99).

The phenol-sulfuric acid method is widely used to quantify total carbohydrate content (TCC) in test samples due to its simplicity, sensitivity, and ability to identify a wide range of carbohydrate types. The principle of the PSA assay involves the dehydration of carbohydrates by sulfuric acid (H_2_SO_4_) (Sigma Aldrich) to produce uronic acid and hydroxyurea formaldehyde. The latter then reacts with phenol (Ambion) to generate a reddish-orange colour, which can be quantified by measuring the absorbance at 490 nm. The intensity of the colour is directly correlated with the concentration of carbohydrates present in the sample [29]. The total carbohydrate content (TCC) of oligosaccharides extracted from ethanol-precipitated polysaccharides of okra pods and seed mucilage, subsequently digested with *P. kudriavzevii* and fractionated via GPC, is demonstrated in Fig 2. Within the obtained fractions, ODF91 exhibited the highest TCC of 6.19 ± 0.06 mg/mL. The findings demonstrate that *P. kudriavzevii* efficiently fermented okra-derived EPP, as no carbohydrates were detected in fractions ODF1 to ODF30, while minimal TCC was observed in fractions ODF31 to ODF50. Besides ODF91, other noteworthy fractions include ODF84, which contained 5.66 ± 0.06 mg/mL TCC, followed by ODF73 (5.13 ± 0.02 mg/mL), ODF78 (4.14 ± 0.09 mg/mL), ODF75 (4.09 ± 0.42 mg/mL), and ODF71 (4.11 ± 0.13 mg/mL). Size-exclusion chromatography (SEC) is a technique that separates oligosaccharides based on their molecular size, allowing the isolation of fractions with different molecular weight distributions that can affect biological activity. In the current research, SEC was used to isolate bioactive fractions, with ODF91 having the greatest biological activity. According to the total carbohydrate content and further compositional profiling, the selected fractions, such as ODF71, ODF83, ODF84, and ODF91, were prioritized to undergo biological screening. These fractions were carbohydrate-rich and compositionally distinct, allowing comparison of their bioactivity. A chromatographic profile (SEC chromatogram) was not included in the present study and will be considered in future investigations for detailed fraction characterization; additionally, future studies should include the determination of protein and moisture content of the extracted EPP.

### 3.3. Monosaccharide composition of the oligosaccharide fractions

**Fig 3:**
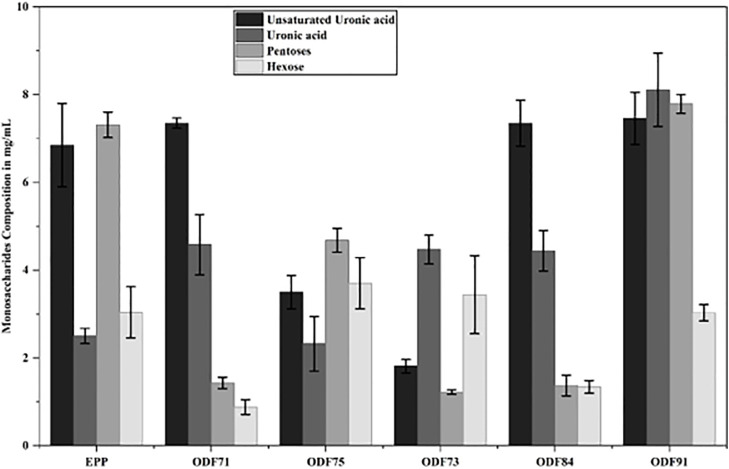
Monosaccharide composition (mg/mL) of ethanol-precipitated polysaccharides (EPP) and oligosaccharide fractions (ODF71-ODF91).

The monosaccharide composition of okra fractions was quantified to assess the contents of uronic acids, unsaturated uronic acids, pentoses, and hexoses. Fraction ODF91 exhibited the highest contents of unsaturated uronic acid (7.46 mg/mL), closely followed by ODF84 (7.35 mg/mL) and ODF71 (7.35 mg/mL), while ODF73 contained a significantly lower amount (1.81 mg/mL) (Fig 3). The overall uronic acid contents were also highest in ODF91 (8.11 mg/mL), with moderate levels in ODF71 (4.58 mg/mL) and ODF73 (4.58 mg/mL). Regarding pentoses, EPP showed the highest concentration (7.31 mg/mL), followed by ODF75 (4.68 mg/mL) and ODF91 (3.79 mg/mL). The hexose contents varied significantly among fractions, with ODF75 containing the highest concentration (3.7 mg/mL), closely followed by ODF73 (3.44 mg/mL) and EPP (3.04 mg/mL). These variations in monosaccharide composition suggest distinct structural characteristics of the oligosaccharide digested fractions purified from okra pods and seed mucilage via microbial digestion, potentially influencing their biological applications. *Pichia pastoris*-mediated digestion of water-soluble polysaccharides from cress seed mucilage produced oligosaccharides with galacturonic acid, glucuronic acid, arabinose, rhamnose, and glucose as primary monosaccharide units [30]. It is important to note that the present biochemical analyses provide preliminary compositional insights into the oligosaccharide fractions. Detailed structural characterization, including molecular weight distribution, linkage analysis, and advanced spectroscopic techniques such as NMR, was beyond the scope of the study and warrants further investigation.

### 3.4. Hemolytic potential of oligosaccharide fractions

**Fig 4:**
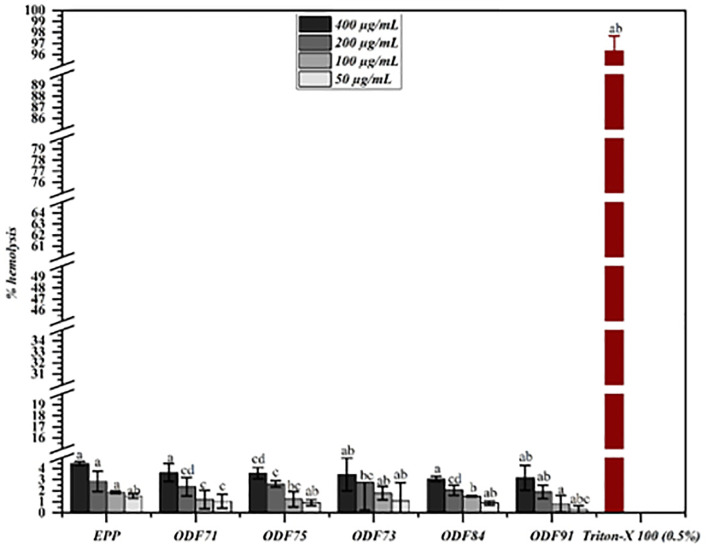
Percent hemolysis induced by oligosaccharides in human red blood cells (HRBCs) at different concentrations (50-400 µg/mL). EPP: ethanol-precipitated polysaccharides, ODF71-ODF91 are oligosaccharide fractions, and Triton-X 100 (0.5%) is a standard hemolytic drug. Data represent the mean ± SD (n = 3), with different letters above indicating significant differences between the groups (p < 0.05).

Hemolysis is critical for evaluating the biocompatibility of substances intended for biomedical applications, as it reflects the potential to disrupt RBC membranes [31]. The hemolysis assay was performed to assess the potential cytotoxicity of oligosaccharide fractions purified from EPP by microbial digestion of okra pod and seed mucilage. Percent hemolysis for each fraction was calculated and presented as mean ± standard deviation for three independent replicates. The results in Fig 4 showed that none of the oligosaccharide fractions induced significant hemolysis at the tested concentrations, including 50 µg/mL, 100 µg/mL, 200 µg/mL, and 400 µg/mL. Compared to oligosaccharide fractions, EPP exhibited the highest hemolytic activity, with 4.24% at 400 µg/mL, close to the 5% safety threshold but still within the acceptable limits. These results confirm the biocompatibility of oligosaccharides, as they exhibit no or very low cytotoxicity in erythrocytes, indicating their safety for biomedical applications. Our findings are consistent with prior studies on plant-derived polysaccharides, which have generally demonstrated very low hemolytic activity and high biocompatibility [32]. It is worth noting that the hemolysis limit for potential biomedical applications is set at <5%. Any increase above this limit is considered toxic and unsuitable for biomedical applications [33]. There was no statistically significant hemolysis (p > 0.05) of any oligosaccharide fraction relative to the negative control, indicating good biocompatibility.

### 3.5. In vitro anti-inflammatory potential of oligosaccharide fractions

**Fig 5:**
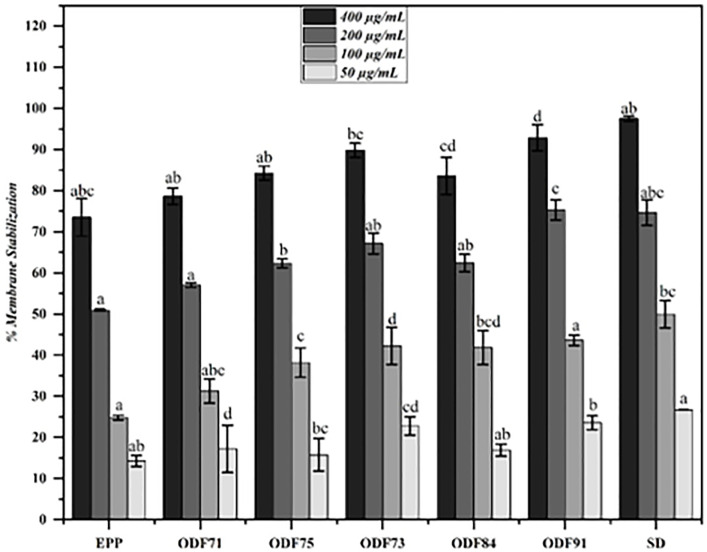
Percent membrane stabilization potential of oligosaccharides at different concentrations (50–400 µg/mL). EPP: ethanol-precipitated polysaccharides; ODF71–ODF91: oligosaccharide fractions; indomethacin: standard drug. Data are expressed as mean ± SD (n = 3). Different letters above the bars indicate statistically significant differences between groups (p < 0.05.

The HRBCs membrane stabilization assay is a well-defined in vitro approach to assess the anti-inflammatory potential of various compounds by measuring their ability to prevent HRBCs lysis under hypotonic conditions. Membrane stabilization is crucial because it mimics the protection of the lysosomal membrane in vivo, thereby preventing the excretion of pro-inflammatory mediators such as proteases and other enzymes that exacerbate inflammation [34]. The current study elucidated oligosaccharides’ dose-dependent membrane stabilization effects, benchmarked against indomethacin, the reference drug. At a 400 µg/mL concentration, ODF91 demonstrated notable erythrocyte membrane stabilization (92.86 ± 3.15%), followed by ODF83 (89.79 ± 1.72%), ODF75 (84.25 ± 1.67%), ODF84 (83.58 ± 4.51), ODF71 (78.63 ± 2.02%), and EPP (73.48 ± 4.54%) as depicted in Fig 5. For comparison, indomethacin exhibited a stabilization percentage of 97.48 ± 0.66%. Moreover, ODF91 showed the most potent EC_50_ value (109.52 ± 3.13 µg/mL), followed by ODF83 (119.49 ± 4.12 µg/mL) and ODF84 (136.28 ± 1.09 µg/mL), as shown in Table 1. Membrane stabilization is a process in which the reliability of the cell membrane, including erythrocytes and lysosomal membranes, is sustained in opposition to osmotic and heat-induced disintegration [35]. The literature suggests that free radical-induced destruction of the RBC membrane is the major source of the decreasing capability of erythrocytes to resist mechanical and osmotic stress [36]. ODF91 exhibited significantly higher membrane stabilization compared to other fractions (p < 0.05), while all fractions showed statistically significant improvement compared to the untreated control. These findings suggest that oligosaccharide fractions may protect erythrocyte membranes against hypotonic stress.

**Table 1:** EC_50_ values (µg/mL) for HRBC membrane stabilization and IC_50_ values (µg/mL) for α-amylase inhibition, α-glucosidase inhibition, and yeast glucose uptake assays of oligosaccharide fractions.

	EC_50_ (µg/mL) HRBC membrane stabilization	IC_50_ (µg/mL) α-Amylase inhibition	IC_50_ (µg/mL) α-Glucosidase inhibition	IC_50_ (µg/mL) Yeast Glucose Uptake
***EPP***	192.95 ± 2.45	185.37 ± 1.93	198.52 ± 2.67	210.64 ± 3.87
***ODF71***	161.09 ± 1.76	92.95 ± 3.18	212.99 ± 1.06	257.47 ± 1.95
***ODF75***	140.86 ± 3.01	186.40 ± 2.23	213.61 ± 1.65	277.66 ± 0.84
***ODF83***	119.49 ± 4.12	204.48 ± 4.12	146.56 ± 2.34	214.90 ± 1.54
***ODF84***	136.28 ± 1.09	86.19 ± 2.09	148.03 ± 1.72	149.32 ± 3.27
***ODF91***	109.52 ± 3.13	85.74 ± 0.65	122.69 ± 2.38	126.82 ± 2.98
***Std. Drug***	99.05 ± 2.76	54.83 ± 1.52	113.33 ± 1.87	109.09 ± 0.45

### 3.6. In vivo anti-inflammatory potential of oligosaccharide fractions

**Fig 6:**
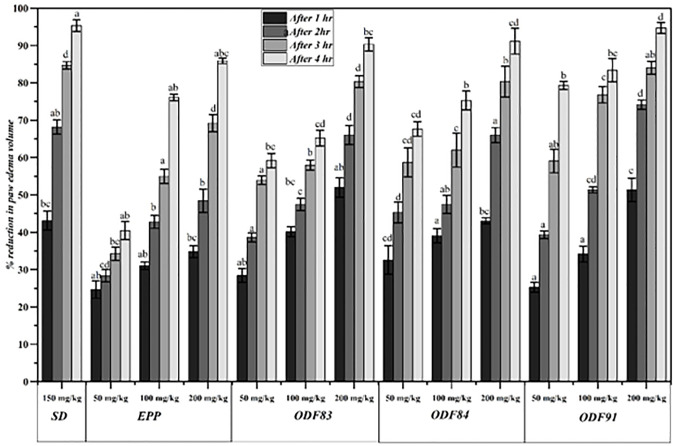
Percent reduction in paw edema volume at different time intervals (1 to 4 h) following administration of EPP: ethanol-precipitated polysaccharides and oligosaccharide fractions (ODF83, ODF84, and ODF91) in the carrageenan-induced paw edema model. SD refers to diclofenac sodium, a standard drug. Data are expressed as the mean ± SD (n = 5), with different letters above indicating significant differences between the groups (p < 0.05).

Building on the promising in vitro anti-inflammatory effects of the oligosaccharide fractions, we sought to investigate their in vivo anti-inflammatory potential. Inflammation is a vital immune response involving multiple cellular processes designed to address tissue injury or infection. This complex reaction is orchestrated by different cell types that respond to a range of signals in a highly regulated manner [37]. Typically, inflammation is characterized by increased production of cytokines and chemokines, which facilitate the migration of leukocytes to the site of damage. However, excessive or chronic inflammation is implicated in several clinical conditions, including psoriasis, rheumatoid arthritis, and inflammatory bowel diseases [38]. To assess the in vivo anti-inflammatory effects of the oligosaccharides isolated from EPP via microbial digestion, the carrageenan-induced paw edema model was employed in mice. Inflammation and edema were induced by the injection of 1% carrageenan into the right hind paw. The data, illustrated in Fig 6, show the percent reduction in paw edema volume, relative to diclofenac sodium (150 mg/kg), which served as the reference. After 4 hours, diclofenac sodium demonstrated a 92.32 ± 1.55% reduction in paw edema, confirming its strong anti-inflammatory action in vivo. Among the oligosaccharide fractions, ODF91 at 200 mg/kg exhibited a remarkable 94.69 ± 1.47% reduction in paw edema at 4 hours, comparable to diclofenac sodium’s efficacy. Additionally, ODF83 and ODF84 at 200 mg/kg also showed significant reductions in paw edema, with values of 90.3 ± 1.75% and 91.14 ± 3.45%, respectively, after 4 hours. A statistically significant reduction in paw edema (p < 0.05) was observed for all treated groups compared to the negative control, with ODF91 showing the most pronounced effect at 200 mg/kg.

The mechanism underlying carrageenan-induced inflammation is well established and occurs in three distinct phases. In the initial phase, following carrageenan injection, serotonin and histamine are released within the first hour. In the second phase, after 2 hours, kinins are produced. In the final phase, after 3 hours, cyclooxygenase (COX) is activated, leading to the synthesis of prostaglandins, which contribute to increased paw edema and inflammation [39]. Moreover, inflammation is associated with the generation of reactive oxygen species (ROS), which further exacerbate edema [40]. Previous studies have shown that polysaccharides from Caesalpinia ferrea and Azadirachta indica exhibit anti-inflammatory effects by reducing the edematogenic impact of serotonin, prostaglandins, and nitric oxide [41,42]. Furthermore, research on polysaccharides extracted from Sedum dendroideum demonstrated their ability to reduce inflammation by lowering TNF-α and IL1-β levels [43]. These findings represent functional screening evidence rather than direct mechanistic validation. Further research is needed to assess the presence of inflammatory mediators, including TNF-2, IL-6, COX-2, prostaglandins, and oxidative stress markers, to validate the pathways.

### 3.7. Percent alpha-amylase inhibition potential of oligosaccharide fractions

**Fig 7:**
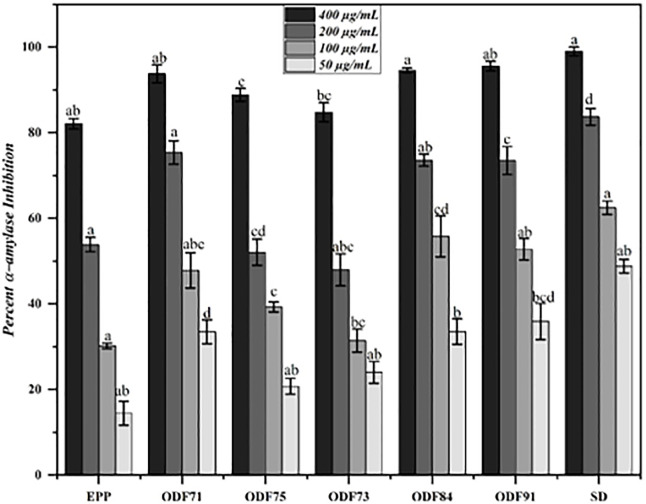
Percent α-amylase inhibition of oligosaccharides derived from EPP through microbial fermentation at different concentrations (50-400 µg/mL). EPP: ethanol-precipitated polysaccharides, ODF71-ODF91 are oligosaccharide fractions, and SD: Acarbose as the standard drug. Data represent the mean ± SD (n = 3), with different letters above indicating significant differences between the groups (p < 0.05).

Alpha-amylase is a carbohydrate-degrading enzyme that digests polysaccharides into monosaccharides [44]. Thus, limiting glucose production from carbohydrate sources by inhibiting carbohydrate-degrading enzyme activities through the consumption of alpha-amylase inhibitors may offer a better strategy for the management of hyperglycemia and T2DM [45]. In the current investigation, we explored the α-amylase inhibitory potential of okra oligosaccharide fractions at three distinct concentrations. Results shown in Fig 7 revealed a dose-dependent inhibition of α-amylase for each fraction, with acarbose used as the standard drug. At 400 µg/mL, fraction ODF91 exhibited 95.54 ± 1.15% inhibitory effect on α-amylase, followed by ODF84 (94.58 ± 0.52%), ODF71 (93.76 ± 2.08%), ODF75 (88.81 ± 1.51%), and ODF73 (84.77 ± 2.24%), while acarbose showed inhibition of 98.99 ± 0.89%. In terms of half-maximal effective concentration (EC_50_) values, the maximum efficacious value was recorded for ODF91 (85.74 ± 0.65 µg/mL), followed by ODF84 (86.19 ± 2.09 µg/mL) and ODF71 (92.95 ± 3.18 µg/mL), as depicted in Table 1. The α-amylase inhibitory effect of oligosaccharides from okra pod mucilage showed better results than polysaccharides extracted from *Cucurbita moschata,* where the activity of α-amylase was inhibited by 41.3% [46]. A previous study demonstrated that polysaccharides purified from *Nitraria retusa* inhibited α-amylase in a concentration-dependent manner, with an IC_50_ of 4.55 mg/mL [42], less potent than our results, where the IC_50_ of fraction ODF91 was 0.085 mg/mL. The basic mechanism underlying most α-amylase inhibitors is that they can reduce glucose levels within the small intestine, further delaying postprandial hyperglycemia [47]. The α-amylase inhibitory response of polysaccharides is associated with their monosaccharide composition, as polysaccharides having the highest uronic acid content exhibit a strong ability to bind with α-amylase [48]. ODF91 demonstrated significantly greater inhibitory activity than other fractions (p < 0.05), indicating its strong potential for α-amylase inhibition. Our results show that fractions containing the highest uronic acid content significantly inhibited α-amylase activity.

### 3.8. Percent α-glucosidase inhibition potential of oligosaccharide fractions

**Fig 8:**
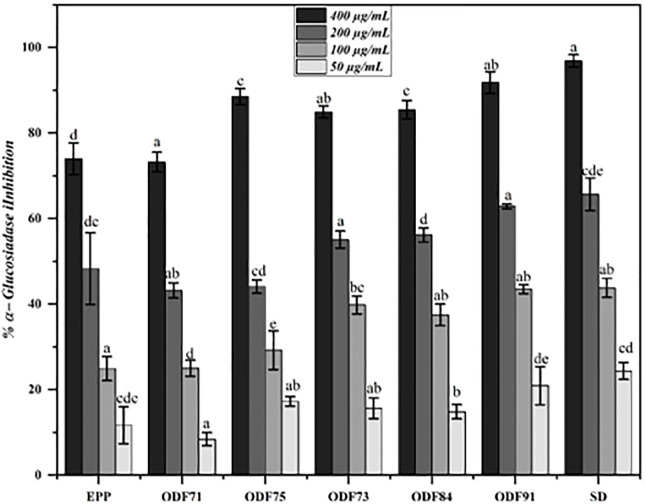
Percent α-glucosidase inhibition of oligosaccharides derived from EPP through microbial fermentation at different concentrations (50-400 µg/mL). EPP: ethanol-precipitated polysaccharides, ODF71-ODF91 are oligosaccharide fractions, and SD: Acarbose, a standard drug. Data represent the mean ± SD (n = 3), with different letters above indicating significant differences between groups (p < 0.05).

α-glucosidase mediates the hydrolysis of terminal non-reducing α-1 → 4 oligosaccharide or disaccharide linkages to produce sugar contents; thus, lowering α-glucosidase production could significantly reduce the amount of glucose released after meals [49]. Alpha-glucosidase inhibition, which significantly lowers postprandial blood glucose levels, was considered an essential component for targeting type 2 diabetes [50]. The in vitro hypoglycemic action of various bioactive polysaccharides has been investigated using glucosidase activity suppression assays [51]. The in vitro antidiabetic efficacy of selected okra polysaccharide fractions was evaluated by measuring their inhibition of α-glucosidase at four concentrations, along with IC_50_ values, as shown in Table 1. The result shown in Fig 8 demonstrated that at 400 µg/mL, fraction ODF91 significantly inhibited alpha-glucosidase by 91.8 ± 2.56%, which was higher than ODF75, ODF84, ODF73, and ODF71, having the percent inhibition of 88.50 ± 1.87%, 85.45 ± 2.13%, 84.93 ± 1.44%, and 73.19 ± 2.31%, respectively. These findings concluded that ODF91, ODF73, and ODF84 had significant antidiabetic effects via inhibiting alpha-glucosidase. The inhibition observed for ODF91 was statistically significant (p < 0.05) compared to other fractions and the untreated control. Okra polysaccharide fermentation-derived oligosaccharides have been shown to suppress alpha-glucosidase and alpha-amylase in vitro in a dose-dependent manner and can be used to manage type 2 diabetes [52]. Our findings were consistent with prior research, which demonstrated that the monosaccharide content and the molecular size distribution of the polysaccharides were associated with α-amylase and α-glucosidase inhibition activity [53].

### 3.9. Effect of oligosaccharide fractions on the glucose uptake in yeast cells

**Fig 9:**
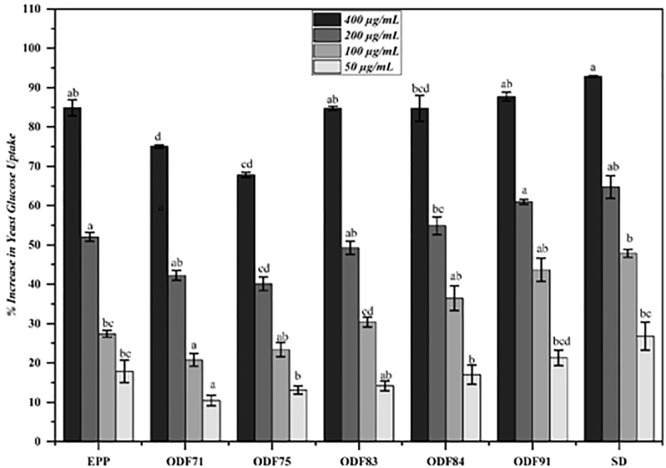
Percent increase in yeast glucose uptake potential of oligosaccharides derived from EPP through microbial fermentation at different concentrations (50—400 µg/mL). EPP: ethanol-precipitated polysaccharides, ODF71-ODF91 are oligosaccharide fractions, and SD: Acarbose is a standard drug. Data represent the mean ± SD (n = 3), with different letters above indicating significant differences between groups (p < 0.05).

The percent increase in glucose uptake by yeast cells from selected okra polysaccharide fractions was evaluated at varying concentrations, and the results are illustrated in Fig 9. At a 400 µg/mL concentration, SD showed glucose uptake up to 92.82 ± 0.25%, while ODF91 exhibited an 87.69 ± 1.13% increase in yeast glucose uptake, followed by EPP (84.86 ± 2.06%) and ODF73 (84.75 ± 0.53%). The lowest glucose uptake was recorded for ODF75 (67.82 ± 0.64%). These results revealed that oligosaccharide fractions obtained from okra mucilage boosted glucose uptake in yeast cells (0–88%) at distinct concentrations. A statistically significant increase in glucose uptake (p < 0.05) was observed for ODF91 compared to other fractions. In yeast (*Saccharomyces cerevisiae*) cells, glucose translocation is intricate, and glucose is transported by facilitated diffusion. Specialized transporters known as “facilitated carriers” carry solutes from higher to lower concentrations, underlining that effective transportation is only feasible if intracellular glucose is eliminated [54]. Therefore, glucose transfer can function independently if intracellular glucose levels are sufficiently reduced. It has been reported that glucose transport across yeast cell membranes may be mediated by facilitated diffusion and that its uptake can be modulated by several factors, including glucose concentration within yeast cells and its subsequent metabolism [23,55]. The tested fraction showed a marked effect on glucose absorption, suggesting it can improve glucose utilization and thereby regulate blood sugar levels [56]. Compared with previous studies, the percent increase in yeast cell glucose uptake observed in the current study may be attributable to oligosaccharides present in okra pods and seed mucilage, obtained through microbial digestion.

### 3.10. Percent reduction in blood glucose level in diabetic mice

**Fig 10:**
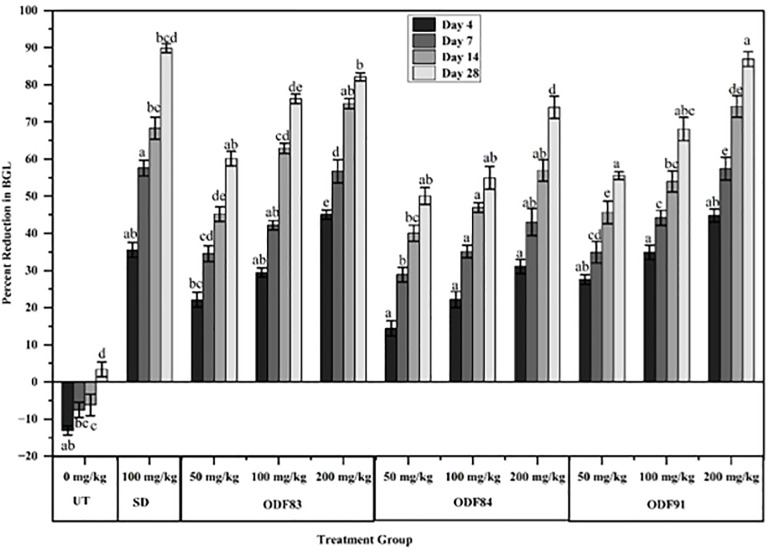
Percent reduction in blood glucose levels (BGLs) at different time intervals (4 to 28 days) following administration of EPP: ethanol-precipitated polysaccharides and oligosaccharide fractions (ODF83, ODF84, and ODF91) in the STZ-induced diabetic mice model. SD refers to glibenclamide (standard drug), and UT refers to untreated diabetic control. Data are expressed as the mean ± SD (n = 5), with different letters indicating significant differences between groups (p < 0.05).

Persistent hyperglycemia generates free radicals that damage pancreatic tissues and islet cells, increasing the risk of diabetes. Scavenging these free radicals will neutralize oxidative stress, which could help manage blood glucose levels [57]. STZ is widely used to induce diabetes in mice because it selectively targets and disrupts pancreatic β-cells. The percent reduction in BGLs in STZ-induced diabetic mice following treatment with different concentrations of oligosaccharides attained through microbial digestion of okra pods and seeds mucilage polysaccharides is illustrated in Fig 10. Diabetic mice administered with 100 mg/kg of glibenclamide (standard drug) demonstrated a significant reduction in BGLs, i.e., 35.52 ± 1.99%, 57.61 ± 2.12%, 68.31 ± 2.12%, and 89.86 ± 1.19% on days 4, 7, 14, and 28, respectively. Among the tested oligosaccharide fractions, ODF91 exhibited the highest reduction on day 28 (86.91 ± 1.99%) at 200 mg/kg, closely followed by ODF83 (82.16 ± 1.06%) and ODF84 (73.93 ± 3.01%). The results also revealed that lower concentrations of oligosaccharide fractions also exhibited a considerable reduction in BGLs. For instance, ODF91 at 100 mg/kg and 50 mg/kg lowered BGL by 68.08 ± 3.13% and 55.54 ± 1.02%, respectively, on day 28. Similarly, at 100 mg/kg and 50 mg/kg, ODF83 exhibited a strong decrease in BGL that is 76.25 ± 1.31% and 60.11 ± 1.95% on day 28, respectively. In the present study, we observed a substantial reduction in BGL similar to our previous study, in which cress seed mucilage polysaccharide digested fractions DF73, DF53, and DF72 reduced BGL by 91.5%, 85.10%, and 78.63%, respectively [30]. Other previous studies also reported an antihyperglycemic effect of polysaccharides in a streptozotocin-induced diabetic mouse model [58]. Similarly, polysaccharides from *Lycium barbarum* showed a strong antihyperglycemic effect in streptozotocin-induced diabetic mice. The antihyperglycemic effect may be due to its enhancement of intestinal glucose absorption and metabolism, improving insulin secretion and sensitivity, and preventing damage to β-cells of the islets of Langerhans [59]. Further studies are required to elucidate the molecular mechanism underlying these effects.

### 3.11. Effect of oligosaccharide fraction on the weight of STZ-induced diabetic mice

**Fig 11:**
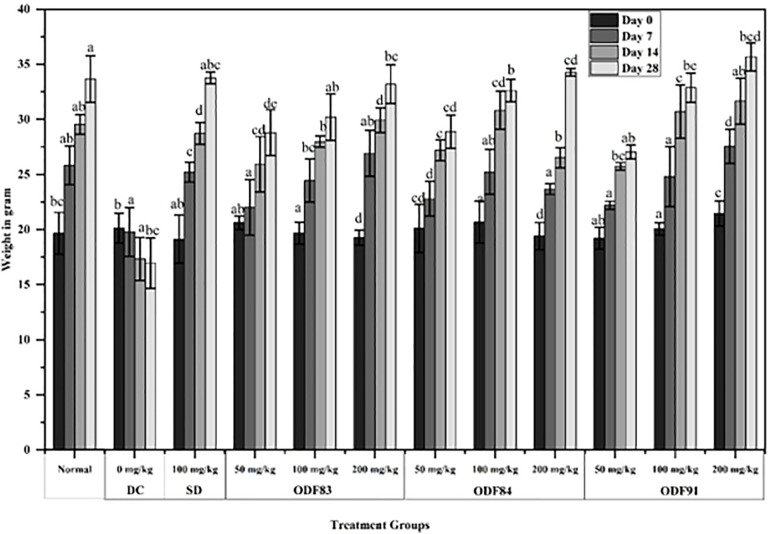
Weight in grams of STZ-induced diabetic mice at different time intervals (4 to 28 days) following administration of EPP: ethanol-precipitated polysaccharides and oligosaccharide fractions (ODF83, ODF84, and ODF91). SD refers to glibenclamide (standard drug), and DC refers to untreated diabetic control. Data are expressed as the mean ± SD (n = 5), with different letters indicating significant differences between the groups (p < 0.05).

Excessive weight loss is often associated with type II diabetes mellitus and can be attributed to enhanced breakdown of muscle and tissue proteins due to impaired blood glucose metabolism [60]. The effects of different concentrations of selected oligosaccharides on the body weight of STZ-induced mice over 28 days are portrayed in Fig 11. The results revealed that untreated diabetic mice showed a decrease in weight over 28 days, from 20.13 ± 1.36 g on day 4 to 16.95 ± 2.28 g on Day 28. Conversely, the group administered 100 mg/kg of glibenclamide exhibited a marked increase in body weight, from 19.12 ± 2.19 g on Day 4 to 33.76 ± 0.53 g on Day 28. Among the screened oligosaccharide fractions, ODF91 at 200 mg/kg resulted in a substantial increase in body weight, i.e., from 21.45 ± 1.13 g on day 4 to 35.67 ± 1.28 g on day 28. Likewise, at 200 mg/kg, ODF83 and ODF84 led to body weights of 33.18 ± 1.76 g and 34.28 ± 0.33 g, respectively, on day 28. Results also showed that lower concentrations of oligosaccharides demonstrated a considerable increase; for instance, ODF83 at 100 mg/kg increased body weight from 19.67 ± 1.01 g on day 4 to 30.19 ± 2.09 g on day 28. These results indicated that oligosaccharides increased the body mass of diabetic mice, with higher concentrations showing a more pronounced effect. Treated diabetic mice showed a significant improvement in body weight compared with untreated diabetic controls (p < 0.05).

### 3.12. Effect of oligosaccharide treatment on dyslipidemia in STZ-induced diabetic mice

**Fig 12:**
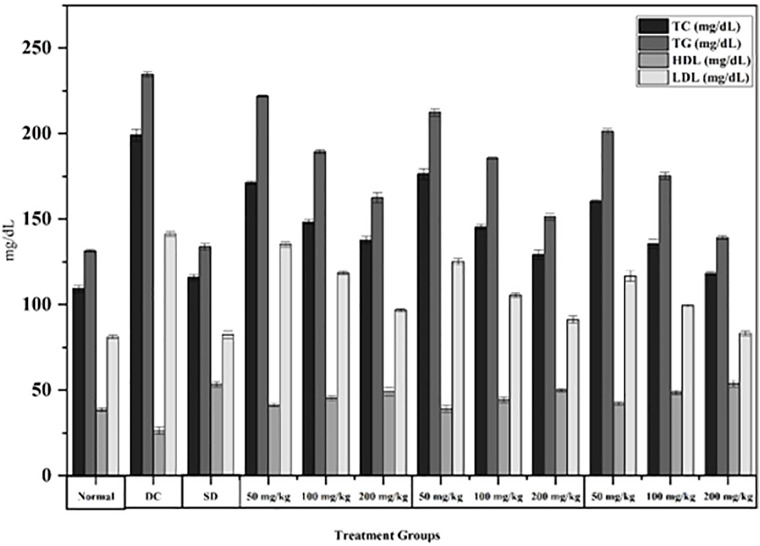
Effect on blood lipid profile of STZ-induced diabetic mice measured at specific time points (Day 4, 7, 14, and 28) following administration of EPP: ethanol-precipitated polysaccharides and oligosaccharides fractions (ODF83, ODF84, and ODF91). SD refers to glibenclamide (standard drug), and DC refers to untreated diabetic control. Data are expressed as the mean ± SD (n = 5).

Insulin resistance and deficiency impair enzymes and pathways involved in lipid metabolism, leading to dyslipidemia [61]. Dyslipidemia, characterized by an abnormal lipid profile, is one of the main complications related to diabetes mellitus. The key changes induced by diabetic dyslipidemia increase LDL, TG, and TC levels and decrease HDL levels. Several studies have reported that inflammation plays a crucial role in the development of diabetes complications [62]. In this investigation, we evaluated the effect of oligosaccharide fractions obtained from microbial digestion of EPP from okra pod and seed mucilage on the blood lipid profile of STZ-induced diabetic mice. The results revealed that STZ significantly reduced HDL levels in mice to 26.31 ± 2.12, compared with normal mice, in which HDL levels were 38.53 ± 1.09 (Fig 12). However, oligosaccharide treatment significantly increased HDL levels in a concentration-dependent manner. Among fractions, ODF91 at 200 mg/kg increased HDL levels to 53.8 ± 2.09, which was comparable to glibenclamide (53.28 ± 1.56), a standard antidiabetic drug.

Furthermore, it was observed that STZ treatment induced a sharp increase in LDL levels (141.29 ± 1.34) compared to those in normal control (81.20 ± 0.92), in which no diabetes was induced. However, at 200 mg/kg, ODF91 prominently decreased LDL levels to 83.29 ± 1.37, followed by ODF84 (91.23 ± 2.31) and ODF83 (96.75 ± 1.04). STZ administration also increased the TG and TC levels in mouse models to (234.54 ± 1.54 and 199.12 ± 3.43, respectively) compared to normal control (131.43 ± 0.69 for TG and 109.24 ± 2.13 for TC). The oligosaccharide treatment significantly restored these abnormalities. ODF91 reduced TG to 139.35 ± 1.07 and TC to 118.27 ± 1.73 at 200 mg/kg, levels comparable to those of glibenclamide (133.76 ± 2.01 for TG and 115.87 ± 1.78 for TC, respectively) (Fig 12). These results confirm that oligosaccharides protect against lipid metabolic disorders in STZ-induced mouse models. Oligosaccharide treatment significantly improved lipid profile parameters compared with diabetic control mice (p < 0.05).

### 3.13. Conclusion

In conclusion, *Pichia kudriavzevii*-mediated fermentation successfully converted okra mucilage polysaccharides into bioactive oligosaccharides. Among the fractions, ODF91 exhibited the strongest anti-inflammatory and antidiabetic activities in both in vitro and in vivo models. These findings highlight the potential of fermented okra-derived oligosaccharides as functional bioactive compounds. However, further structural characterization and mechanistic studies are required to support their application. Future research should focus on mechanistic pathways, feasibility of scaling up, safety evaluation, and clinical validation to support the development of these oligosaccharides as promising nutraceuticals.

## Supporting information

S1 Checklist Inclusivity in global research. (DOCX)
